# A Comprehensive Bibliometric Study in the Context of Chemical Hazards in Coffee

**DOI:** 10.3390/toxics12070526

**Published:** 2024-07-22

**Authors:** Grobert A. Guadalupe, Dorila E. Grandez-Yoplac, Ligia García, Eva Doménech

**Affiliations:** 1 Instituto de Investigación, Innovación y Desarrollo para el Sector Agrario y Agroindustrial de la Región Amazonas (IIDAA), Universidad Nacional Toribio Rodríguez de Mendoza de Amazonas, Chachapoyas 01001, Peru; yoplacesteffany@gmail.com; 2 Instituto Universitario de Ingeniería de Alimentos Food-UPV, Universitat Politècnica de València, Camino de Vera s/n, 46022 Valencia, Spain; 3 Instituto de Investigación para el Desarrollo Sustentable de Ceja de Selva (INDES-CES), Universidad Nacional Toribio Rodríguez de Mendoza de Amazonas, 342 Higos Urco, Chachapoyas 01001, Peru; ligia.garcia@untrm.edu.pe

**Keywords:** risk assessment, pollutants, health, environment, coffee by-products

## Abstract

The research aimed to carefully review the chemical hazards linked to the coffee production chain to analyse the risks and opportunities for consumers and the environment, as well as identify potential knowledge gaps. The Scopus database was consulted from 1949 to April 2024 to conduct a bibliometric analysis. As a result, 680 articles were analysed. Results indicated a significant increase in research activity since 2015. China, Brazil, and the USA were the leading countries in scientific production and collaborations. The most prolific journals in this field were *Chemosphere*, *Science of the Total Environment*, *Food Chemistry*, *Journal of Agricultural* and *Food Chemistry*, and *Journal of Environmental Management*, all of which are in the first quartile. The word analysis revealed two main themes: the first focuses on the chemical hazards of coffee and their impact on health, while the second explores the waste generated during coffee production and its potential for reuse. The topics covered in the research include the composition of coffee, associated chemical hazards, possible health risks, and ways to reuse waste for environmental protection. Future research should concentrate on optimising techniques and processes to ensure quality, safety, and sustainability.

## 1. Introduction

Coffee is a highly popular beverage consumed all around the world. In the 2021/22 period, coffee consumption has increased by 4.2%, with 175.6 million bags of coffee being consumed. The most commonly grown variety is Arabica coffee, with a production of 98.6 million bags in 2022/23, and it is also the most consumed type due to its exceptional taste and aroma. Therefore, coffee cultivation has a significant economic impact on the countries with the highest production, such as Brazil, Colombia, Ethiopia, Honduras, and Peru. [1].

The process of coffee production starts with the harvesting of coffee beans. Following this step, various layers like the husk, mucilage, and parchment are removed to extract the green bean, which moves on to the roasting stage. The roasting also eliminates the thin silver skin enveloping each bean, resulting in what we recognise as roasted coffee. This coffee is then either directly packaged, ground, or further processed into products such as coffee powder or decaffeinated coffee. Throughout these stages, from harvesting to processing and ultimately to consumption, over two million tonnes of diverse waste materials, including coffee husks, leaves, and grounds, are produced each year [2]. These wastes contain organic compounds like caffeine, tannins, and chlorogenic acid, which could potentially harm the environment if not properly managed [3]. On a more positive note, coffee waste is rich in valuable components such as lipids, cellulose, hemicellulose, polyphenols, carbohydrates, and proteins, among others. As Hoseini and co-authors pointed out in 2021, these substances can be transformed into beneficial products, including biofuels, biochar, dietary fibres, flavourings, bioactive compounds, and carotenoids, thereby offering a sustainable solution to the waste management issue in coffee production.

Chemical hazards in coffee can arise naturally or be added during food production, processing or transport, and therefore pose a risk to consumers’ health. According to the Rapid Alert System for Food and Feed from 2020–2024 [4], chemicals were the most frequently reported hazards, with ochratoxin A ranking first at 45%. Pesticides were the second most reported hazard at 41%, where chlorpyrifos accounted for 66% of the cases.

In 1991, the FAO/WHO Conference on Food Standards, Chemicals, and the Food Trade suggested that the Codex Alimentarius Commission (CAC) adopt risk analysis principles into their decision-making processes. Risk analysis has been recognised as an essential part of food safety since then, consisting of three basic elements: risk assessment, risk management, and risk communication [5]. In 2009, the FAO/WHO published a guide for chemical risk assessment, reflecting significant advances in chemical analysis, toxicological assessment, and risk assessment procedures. The guide details the principles and methods for assessing the risks of chemicals in food [6].

Bibliometric analysis is an effective quantitative method used for describing, evaluating, and monitoring data. This method provides an overview of studies while predicting research development trends [7,8]. Bibliometric analysis typically consists of two parts: performance analysis and scientific mapping. Performance analysis aims to reveal the contribution of research based on information on regions, topics, journals, and authors of publications. On the other hand, scientific mapping seeks to identify links between topics in the field under study [9].

The objective of this research was to conduct a comprehensive study on the chemical hazards associated with coffee throughout the food chain to analyse the risks and opportunities for consumers and the environment, as well as to identify potential knowledge gaps. With this aim, a bibliometric analysis to identify annual evolution, countries, collaborations, journals, authors, affiliations and keywords was performed (Section 3.1). Chemical hazards were analysed (Section 3.2). Risk assessment was reviewed (Section 3.3). Finally, trends and suggestions were discussed (Section 3.4).

## 2. Materials and Methods

### 2.1. Data Selection

**Figure 1:**
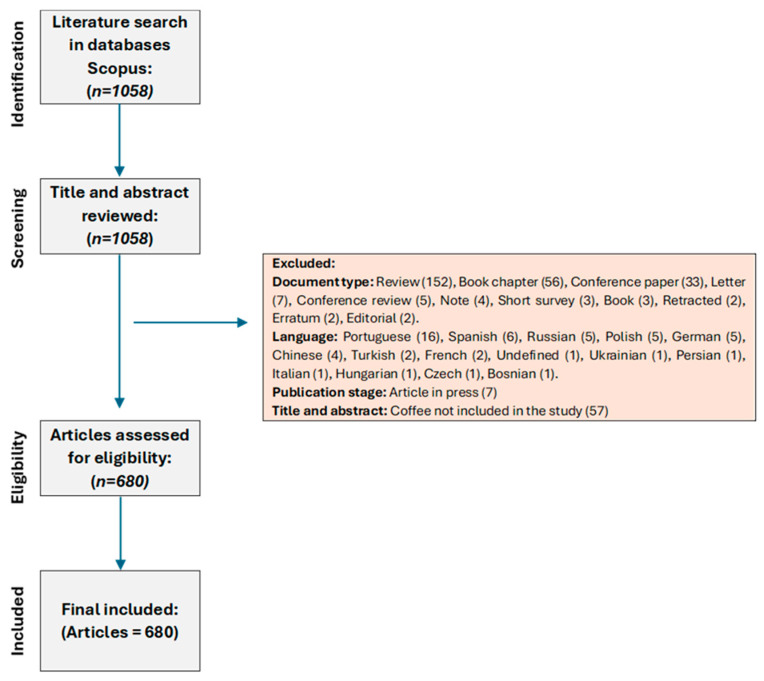
Process of selection papers based on PRISMA methodology.

The search for articles on chemical hazards associated with coffee was performed using the Scopus database, adhering to the PRISMA search methodology [10,11,12,13]. The search field chosen was “Title, Abstract, Keywords” and the keywords used were “coffee” AND “chemical” AND “hazard” OR “risk” OR “contaminant” OR “toxic” OR “pollutant”. The temporal scope of the search was defined from the year 1949 up to 2024 (as of 15 April 2024). To refine the search results, records were filtered to include only “article” type publications that were available in full text, published in English, and at the final stage of publication. This comprehensive search strategy resulted in the selection of 680 documents, constituting the raw data for the present bibliometric analysis, Figure 1.

### 2.2. Subsection

**Figure 2:**
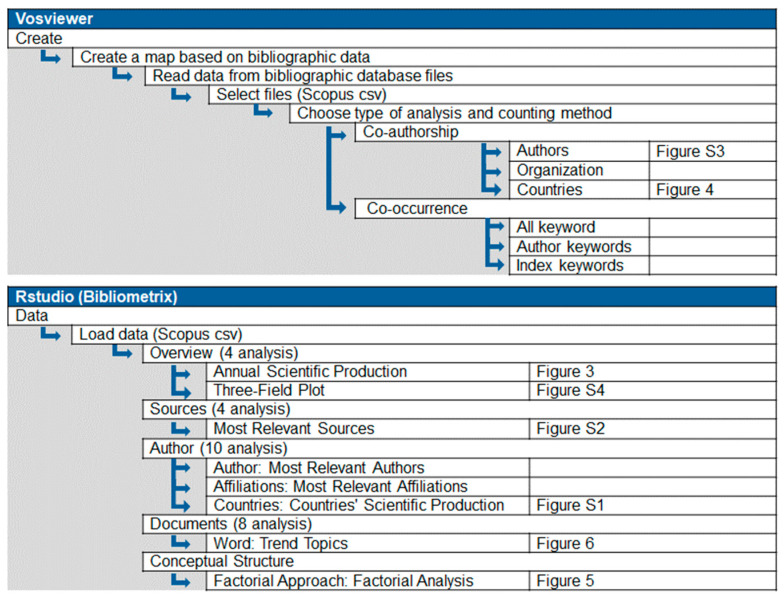
VOSviewer and RStudio (Bibliometrix) stages and studies.

The processing and analysis of the information was carried out using VOSviewer (Version 1.6.20) and the R software combined with the Bibliometrix package (Version 4.3.3). Both programs are based on the “.csv” file obtained from the Scopus database. Figure 2 delineates the various bibliometric analyses possible through these applications, specifically highlighting the ones employed in this study, alongside the resultant figures obtained for each analysis.

## 3. Results and Discussion

### 3.1. Bibliometric Analysis

#### 3.1.1. Annual Scientific Output

**Figure 3:**
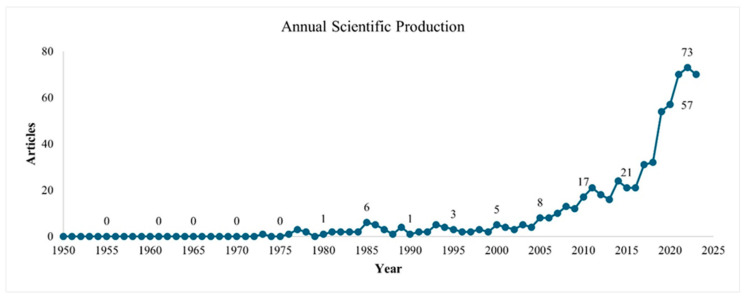
Annual evolution of publications related to hazards and coffee.

The study of the potential hazards associated with coffee began in 1949 with the first recorded publication on the topic. Between 1950 and 1976, research in this area was limited, resulting in just one or no publications per year. From 1977 to 2000, there was a slight increase in activity with between two to six papers published annually. However, it was not until after 2002 that research in this field experienced a substantial surge, particularly from 2015 onwards when the output of research began to grow exponentially. In 2022, the trend peaked, with a record of 73 publications on the subject, highlighting the increasing interest among researchers in the chemical hazards associated with coffee, Figure 3.

#### 3.1.2. Scientific Production by Country

**Figure 4:**
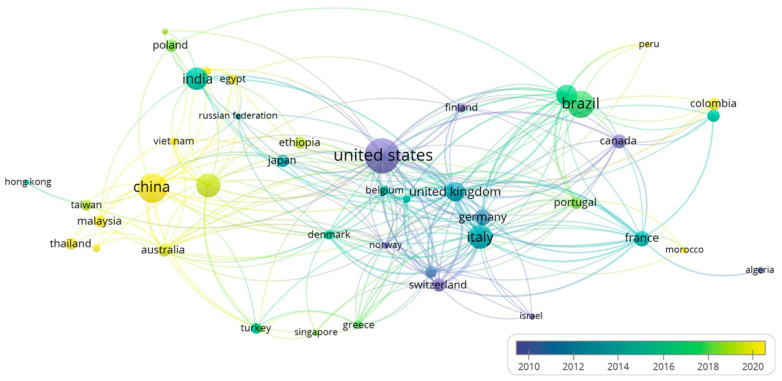
Evolution of publications by country and international collaborations.

Research on chemical hazards in coffee has been conducted across 78 countries. Leading the way in terms of the number of articles produced are China (with 464 publications), Brazil (with 332 publications), and the USA (with 307 publications) (Figure S1). Additionally, Figure 4 provides a visual representation of the countries with the highest publication counts (indicated by larger circle sizes), the evolution of these publications over time (indicated by colour changes), and the collaborative networks between countries (shown as lines connecting circles).

Findings show that, between 2000 and 2010, the USA, Canada, and Switzerland were the most prolific publishers in this domain (represented by dark blue circles). More recently, from 2020 to the present, China and South Korea have emerged as the top contributors (represented by yellow circles). Furthermore, the analysis highlights the significance of international cooperation in advancing scientific knowledge on this topic. The countries with the most significant output (China, the USA, and Brazil) also engage in the most extensive collaboration with international partners. Notably, the USA and Italy have worked together on six documents, while Brazil and Spain, China and South Korea, and China and Australia have collaborated on five documents each. This trend emphasises the global nature of research into chemical hazards in coffee and underscores the importance of cross-border partnerships in fostering scientific progress.

#### 3.1.3. Contribution of the Journals

The journals were evaluated according to the number of publications and the Scimago Journal & Country Rank (SJR) for the year 2023 (SJR, 2024). Out of the 680 papers obtained in Scopus, 357 journals published them, but only 20 journals published more than 5 papers. Figure S2 displays the 20 most significant journals, their quartile, and the number of publications on this topic. The results indicate that 90% (18) of the journals belong to the first quartile and only 10% (2) to the second quartile. *Chemosphere* and *Science of the Total Environment* have the highest number of publications with 20 articles, followed by *Food Chemistry*, *Journal of Agricultural and Food Chemistry*, and *Journal of Environmental Management*, with 17 publications each. The publication of the top five journals has a total of 4799 citations, with an average of 52.7 citations per paper.

#### 3.1.4. Contribution of the Authors

The expertise of an author in a particular field is often determined by the number of scientific articles they have published and the number of citations received for each article. Under this criterion, the most influential author in the field was Zhang, X. with 9 publications and 496 citations, followed by Guo, W. and Liu, Y. with 7 publications, 235 and 67 citations, respectively. It was observed that the majority of the leading researchers in the subject come from China, which is consistent with the country’s progressive growth in scientific output.

Scientific collaboration, or co-authorship, is essential for the advancement of science. Out of 680 articles, the VOSviewer program identified 84 clusters. It revealed that only 18 manuscripts were authored by individuals. Collaboration between two researchers was predominant in 17 clusters, followed closely by collaboration between three researchers in 15 clusters. There were six clusters with four and five researchers, while only a few papers had more than six co-authors (see Figure S3).

#### 3.1.5. Contribution of the Affiliations

A total of 920 affiliations were identified. Among them: Third Military Medical University and Tianjin Chengjian University (China); Federal University of Espirito Santo (Brazil); University of Rome Sapienza (Italy); National Institute for Occupational Safety and Health (USA); and Kangwon National University (Korea) were the most frequently mentioned (Figure S4).

#### 3.1.6. Keyword Analysis

**Figure 5:**
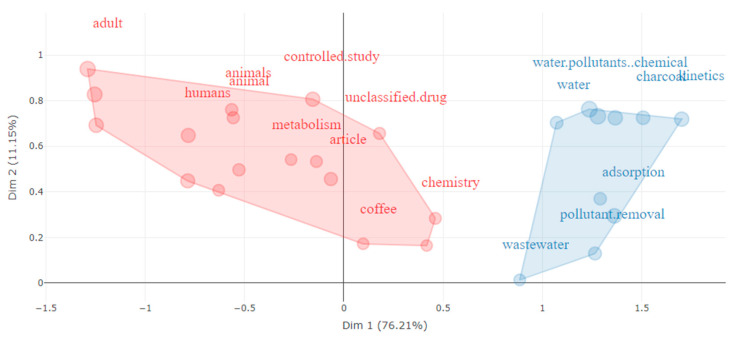
Factorial correspondence analysis of the keyword.

The analysis of the keyword was carried out with the Bibliometrix programme, resulting in 9718 words obtained from the titles, abstracts, and keywords of the publications. By applying a minimum threshold of five co-occurrences, the number of keywords was reduced to 936. Figure 5 displays the factorial correspondence analysis of the keyword. The outcomes reveal that the extracted terms can be grouped into two primary clusters. Cluster 1 (salmon colour) includes keywords such as “controlled study”, “human”, “animal”, “metabolism” and “chemistry”, which were included in work related to chemical hazards in coffee and their impact on consumers’ health. Cluster 2 (light blue colour), on the other hand, comprises words such as “adsorption”, “spent coffee grounds”, “contaminant removal” and “water pollutant”. These terms were used in research focusing on the problem of waste generated by the processing or consumption of coffee and the opportunities resulting from the reuse of these wastes as by-products.

### 3.2. Chemical Hazards in Coffee: From Field to Cup

Coffee is a perennial crop that reaches a constant bean production after 5 to 6 years [14,15]. During this time, it is exposed to natural contaminants from the environment and agricultural practices [16]. One of the most studied contaminants in coffee is metals. The accumulation of these elements in plants has been studied, mainly due to being pulled up by their roots, transported through the xylem, and distributed to aerial sink tissues and fruit [17,18,19,20]. Among the most studied metals in coffee beans are inorganic arsenic (iAs), cadmium (Cd), chromium (Cr), copper (Cu), iron (Fe), mercury (Hg), molybdenum (Mo), nickel (Ni), lead (Pb), and zinc (Zn) [21,22,23,24,25]. Some authors have studied the effects of the coffee-making process on these metals. They concluded that hulling and roasting are the most influential stages, particularly in reducing the levels of chromium (Cr) and lead (Pb). Conversely, the concentration of metals passing from the ground coffee to the coffee beverage is very low [25,26,27,28]. The application of various analytical techniques for improved metal detection has been explored, including the dried droplet method (DDM) with superhydrophobic-induced enrichment, solid-phase extraction followed by spectrofluorimetric analysis, laser-induced breakdown spectroscopy (LIBS), and flame atomic absorption spectrometry (F-AAS) with deuterium background correction [29,30,31,32,33,34]. The International Agency for Research on Cancer has classified several metals as human carcinogens: iAs, Cd, and Ni in Group 1; Pb in Group 2B; and Cr, Cu, Fe, and Hg in Group 3. Non-genotoxic effects have been linked to nephrotoxic and neurotoxic damage, bone fractures, and renal dysfunction, while genotoxic effects have been associated with lung cancer, skin cancer, and cardiovascular issues [35].

Pesticides are commonly used in coffee cultivation to prevent diseases and control pests. Consequently, coffee beans may contain residues that can have adverse health effects [36]. Studies have focused on pesticide concentration in leaves [37], green beans [38,39] and roasted coffee [38,40,41,42,43], as well as the reduction of pesticide concentration during processing. Chen et al., (2019) [36] found that washing coffee beans reduced dinotefuran contents by 44.4% to 86.7%, and the roasting process further reduced these contents by 62.2% to 100%. Additionally, research has been conducted on the development and validation of pesticide analysis methods, such as organochlorines, organophosphates, cypermethrin, permethrin, herbicides, etc., in coffee [44,45,46,47,48]. Some authors have emphasised the significance of the extraction step and the analytical performance of an ionisation source [41,49]. Pesticides are known to be linked to various health issues such as Hodgkin’s and Parkinson’s disease, endocrine disruption, and respiratory, cardiovascular, and reproductive disorders. They can also lead to late neuropathy due to overexposure to organophosphates [50,51,52,53,54,55,56,57,58,59]. Additionally, the use of pesticides in coffee cultivation poses a significant risk to the environment. De Queiroz et al., (2018) [60], concluded that pesticides such as ametryn, cyproconazole, diuron, and triazophos have a 44.7% chance of contaminating surface water and a 23.7% chance of contaminating groundwater. Sulfentrazone and thiamethoxam were also found to have the potential to leach into groundwater [61]. García Ríos et al., (2020) [62] discovered pesticide residues in 81.3% of water samples, with 4,4′-DDT (38%), endosulfan II (19.7%), endosulfan sulphate (11.7%), and endrin (8.8%) being the pesticides most commonly found in surface water.

After the grains have been harvested, improper storage conditions at any point in the food chain can encourage the growth of mycotoxigenic fungi such as *Fusarium*, *Aspergillus ochraceous*, and *Penicillium verrucosum* [63,64,65,66,67]. The secondary metabolism of these fungi produces mycotoxins, with aflatoxins (group 1 carcinogens) and Ochratoxin A (OTA, group 2B) being the most studied in coffee (IARC, 2024). These mycotoxins are also linked to kidney, liver, and neurological damage, among other health issues [68,69,70,71,72,73,74,75,76,77,78,79,80,81,82,83,84,85]. The studies revealed a high presence of OTA in the analysed grains. Pakshir et al., (2021) [74] concluded that 100% of the samples were contaminated, but more than 50% were within the acceptable level. Napolitano et al., (2007) [73] investigated the reduction of OTA contamination during the roasting phase and found that it was below the recommended level of 4 μg/kg. Yassein and Elamary (2021) [83] observed good results regarding the inhibitory effects of five bacterial strains on the growth of *Aspergillus niger.* Additionally, improved analytical methods were applied for fast and efficient determination [76,77,82].

After the pre-harvest process, coffee beans undergo processing. Coffee can be processed in three different ways: dry, semi-dry, and wet processing. In dry processing, the most common system is beans are placed on cement platforms, where they remain for approximately three weeks or until the moisture content reaches 12%, if faster drying is needed, the beans can be subjected to mechanical dryers after exposure to the sun [86]. Dry processing is usually more cost-effective, requiring fewer processing steps and less water. In wet coffee processing, the beans undergo a washing process, followed by peeling, pulping or de-pulping, rinsing, and sun drying [87]. Effluent disposal from pulping, fermentation, and washing of coffee beans contains a high concentration of various substances that are undesirable for discharge into soil or water due to their impact on the environment. These substances may include organic matter, nutrients, salts, agrochemical contaminants, phosphates, nitrates, caffeine, sugars, phenolic compounds, fatty acids, lignin, cellulose, pectic chemicals, and other macromolecules [36,88,89,90,91,92,93,94]. Some authors have studied potential treatments for wastewater in view of its possible reuse for irrigation, recreational use, etc. Hybrid photoelectrochemical oxidation using UV and hydrogen peroxide methods removed nitrates and phosphates by more than 99% [95]. The pulsed electrocoagulation process, combined with defined pH, reaction time, current, and electrolyte concentration, was more than 98% effective in removing chemical oxygen demand, colour, turbidity, phosphate, and nitrate from wet coffee processing wastewater [92,93]. Natural and specially constructed wetland systems were effective in the removal of organic pollutants [96].

During the roasting process, the heat treatment of coffee can result in the production of acrylamide, furans (C_4_H_4_O), polycyclic aromatic hydrocarbons (PAH), and diacetyl. Acrylamide is formed at temperatures above 120 °C. This hazard is considered a by-product of the Maillard reaction, which occurs through the interaction of reducing sugars (especially glucose and fructose) and the amino acid asparagine [97,98,99]. Studies have shown that acrylamide is carcinogenic in rodents and is considered a probable human carcinogen (Group 2A). Additionally, exposure to acrylamide may lead to neurotoxic effects, causing irritation to the eyes, skin, and respiratory tract [100,101,102,103,104,105]. Several studies have examined the concentration of acrylamide in coffee samples [106,107,108]. They have also focused on developing simple and rapid methods for detection and quantification [109], investigating the influence of roasting levels [110], and the impact of cold brewing [111,112]. Other studies have explored the efficacy of asparaginase application for reducing acrylamide formation [113] and the effect of roasting with superheated steam to mitigate its formation [114]. Some research has also reflected on the correlation of acrylamide formation with furans [115] and its relationship with the physicochemical parameters of the beverage [115,116]. Additionally, studies have investigated the influence of coffee variety and agricultural practices [117] and the development of electronic indirect prediction devices [118], dietary exposure [119,120,121,122,123] and consumer risk characterisation [124,125,126]. On the other hand, furan generation is mainly caused by thermal degradation of carbohydrates, oxidation of polyunsaturated fatty acids and decomposition of ascorbic acid or its derivatives [127,128,129,130,131]. IARC classified this compound as a probable Group 2B carcinogen. Furthermore, it is associated with liver cell necrosis, increased hepatocyte proliferation, hepatocarcinogenesis and toxicity in the male reproductive system [132,133,134,135]. In furans, concentration has been evaluated [136,137,138,139,140], development of new technologies with high sensitivity with low detection and quantification limits [118,141,142,143,144], influence of geographical origin and coffee variety [145], correlation with acrylamide [115], kinetics of formation in roasting [110,146], influence of roasting level [147,148], influence of cold brewing [111,112] and consumer risk [137,149]. In coffee, furan mitigation studies have been developed with the incorporation of phenolic acids, flavonoids, non-phenolic antioxidants and non-antioxidant agents [150,151]. Additionally, other chemical hazards produced during heat treatment, such as polycyclic aromatic hydrocarbons (PAH) and diacetyl, have been less frequently studied. PAH are food contaminants that are suspected carcinogens and mutagens, causing oxidative skin damage. Moreover, their oxygenated and nitrated derivatives (which may be more toxic) have been found in some foods [152,153,154,155]. In the field of PAH, more sensitive quantification methods have been evaluated [156,157,158], the concentration in roasted coffee [159,160], the effect of roast level [161]; the effect of roasting with superheated steam to alleviate PAH, acrylamide and acrolein formation [114] and the cancer risk to the consumer [162,163]. Diacetyl is a potentially harmful chemical that is used as an artificial flavouring in the food industry and can also be generated during the processing of some natural products, including coffee. Diacetyl (2,3-butanedione) and 2,3-pentanedione can cause airway epithelial necrosis, damage biological molecules and disrupt protein homeostasis [164]. Consequently, the concentration of diacetyl and the related compound 2,3-pentanedione in the coffee roasting process has been evaluated [165,166,167]. In Europe, a time-weighted average occupational exposure limit of 20 ppb has been adopted for diacetyl and a short-term exposure limit of 100 ppb [168].

Packaging plays a crucial role in preserving food by maintaining its quality and safety [169]. However, the interaction between food and packaging has raised concerns about potential health risks due to the migration of contaminants such as toxic metals [170], alkyl diethanolamines [171], bisphenol A [172,173], dioxins [174], and per- and poly-fluoroalkyl substances [175], trimethyl diphenylmethanes [174], microplastics, phthalates and bisphenol A from paper and plastic cups, when exposed to hot water in coffee consumption [176]. Another important aspect that authors have studied is the environmental impact caused by microplastics generated by disposable plastic cups [177,178,179,180].

The effects of coffee consumption on health have been extensively researched. Daily intake of high doses has been linked to an increased risk of renal cell carcinoma [181], bladder cancer [182], and pancreatic cancer (especially with filtered and boiled coffee) [183]. Additionally, it has been associated with an increased risk of acute coronary syndrome, breast cancer, and colon cancer [184,185]. Furthermore, regular coffee consumption has been linked to increased sperm motility, decreased seminal volume, and decreased semen quality [186]. In contrast, some authors highlight the positive effects of coffee consumption, linking it to a reduced risk of developing type 2 diabetes, since cafestol increases insulin secretion [187]. Additionally, coffee ingestion has a cytoprotective effect [188] and a positive influence on Parkinson’s disease [189].

Throughout the coffee production process, a significant amount of solid waste is produced, including pulp, husk, low-quality beans, silver skin, and coffee grounds after brewing. This waste has a great impact on the environment. Many studies have been conducted to find ways to reuse and add value to this waste. For example, coffee pulp has been studied as a bio adsorbent for removing chromium (VI) [190,191,192,193], As(V) and Pb (II) [194,195], Cd [196], methyl orange [197], methylene blue [198,199,200,201], the herbicide metazachlor [202], crystal violet (cv) dye [203], radioactive waste containing mainly uranium and americium [204], antibiotics [205], etc. Furthermore, coffee husk has been studied for its potential use as a food ingredient due to its phytochemical and antioxidant dietary fibre content [72], as well as for its use as a natural compost [206].

After processing, the low-quality green coffee beans, which would not be marketable, can be reused for the production of a halotolerant biofilm. This biofilm is capable of degrading polynuclear aromatic hydrocarbons and phenanthrene in seawater [207]. The porous structure of the biofilm supports the growth of bacteria capable of degrading organochlorine pesticides [208]. Another potential use is in the biosynthesis of nano-sized heterometallic spinel ZnCo_2_O_4_ particles [209].

The silver skin of coffee is the only residue left after roasting. Studies have found low concentrations of certain substances such as PAH, OTA, pesticides, 5-Hydroxymethylfurfural, chrysene, phenanthrene, fluoranthene, heavy metals, and acrylamide in this residue [72,210,211,212]. However, it has been discovered that the residue has good water and oil retention capacities, emulsion activity and stability, and antioxidant potential. This makes it a potential by-product for use in the food and pharmaceutical industries [72,213]. Additionally, it has shown promising results as a raw material for wood preservatives with antifungal properties [214], as well as an antibacterial agent [215], a biofuel source [216], and as a base for producing biochar capable of absorbing organic pollutants in water [217].

Spent coffee grounds have been used to create biochar that can remove Congo red from water [218], absorb phosphorus [219,220], antibiotics and drugs such as naproxen, diclofenac, ibuprofen, sulfamethoxazole, and tetracycline [221,222,223,224,225]. They have also been effective in adsorbing xenobiotic organic compounds such as paracetamol, diethyltoluamide, bisphenol A, oxybenzone, triclosan, and nonylphenol [226], as well as formaldehyde and other substances [227].

### 3.3. Risk Assessment in Coffee

Out of the 680 scientific papers included in the study, 21 of them conducted a chemical risk assessment on coffee. These papers were written by 107 authors from 21 different countries and were published between 2009 and April 2024. These papers spanned across 17 different journals. Among them, *Biological Trace Element Research* showed the most interest in the topic with three publications, followed by *Foods* and *International Journal of Environmental Analytical Chemistry* with two publications each. The remaining journals only published one article. All the articles were co-authored, with most of them having six researchers involved. International collaboration occurred in 47.6% of the articles. Over the years between 2009 and 2021, there was little variation in the number of articles published, ranging from 0 to 1. However, in 2022, there was a significant increase with five publications, indicating the growing popularity and importance of this research topic worldwide. The study by Kuiper-Goodman et al. (2010) [228] on the topic of probabilistic health risk assessment of Ochratoxin A (OTA) was the most cited paper, with 118 citations.

Table 1 presents information on the chemical hazards, type of coffee studied, risk metrics calculated, equations used, and the approach taken in 21 research papers.

In view of the results, the most studied chemical hazard was acrylamide (nine publications), followed by furan and derivatives and lead (seven), and Ochratoxin A (six publications). Instant coffee was the most studied type of coffee. The metrics used varied depending on the mode of action studied. The Hazard Quotient (HQ) was used to characterise chemicals with a threshold level. The risk was calculated by dividing the exposure to a non-genotoxic chemical hazard by the reference value at which no adverse effects are expected. The result is a numerical value that is considered negligible when it is less than one (Equation (1)).

(1) $$Hazard\;quotient\;(non-dimensional)=\frac{Exposure\;(mg/kgBw/day)}{Reference\;Value\;(mg/kgBw/day)}$$

The risk of non-genotoxic effects can also be obtained by calculating the margin of exposure (MOE) or margin of safety [238]. For risks associated with genotoxic effects, the MOE is adopted by the Joint FAO/WHO Expert Committee on Food Additives and the European Food Safety Authority as an indicator of the level of concern [239,240]. In both cases, for neoplastic and non-neoplastic the MOE is calculated by comparing the defined reference value, usually the BMDL% associated with a percentage increase in the response, to human exposure (Equation (2)). An MOE value of 10,000 or higher is generally considered to be of low concern from a public health perspective.

(2) $$Margin\;of\;exposure\;(non-dimensional)=\frac{Reference\;value\;(mg/kgBw/day)}{Exposure\;(mg/kgBw/day)}$$

Cancer risk is a measure used to evaluate the potential risk associated with exposure to genotoxic and carcinogens during a lifetime. This measure is obtained by multiplying the exposure by a slope factor, Equation (3). A cancer risk lower than 1.0 × 10^−6^ is considered low and does not result in any adverse health effects. Risks between 1.0 × 10^−4^ and 1.0 × 10^−6^ are generally considered moderate, while risk values greater than 1.0 × 10^−4^ are considered unacceptable [241].

(3) $$Cancer\;risk\;(non\_dimensional)=Exposure\;(mg/kgBw/day)\times {slope\;factor\;(mg/kgBw/day)}^{-1}$$

The findings from various studies suggest that, overall, the safety of coffee consumption is high. However, it is important to note that for non-cancerous risks, the lowest margin is associated with iAs, Pb, acrylamide, and furans, while the highest likelihood of cancer is linked to iAs.

The seven columns in Table 1 provide information on whether authors used a deterministic or probabilistic approach. A deterministic approach involves assigning a single value to each parameter in the equation, such as the mean or the 95th percentile, resulting in a single value that represents the risk for a single virtual consumer. Although simple and fast, this method tends to overestimate risk [242]. On the other hand, the probabilistic approach considers the variability and uncertainty of each input parameter, obtaining as a result a probability density function of the risk. Therefore, probabilistic models are better suited to capture the variability and randomness of observed events. This second option was preferred by the reviewed authors.

### 3.4. Trends and Suggestions for Future Research on Chemical Hazards in Coffee

**Figure 6:**
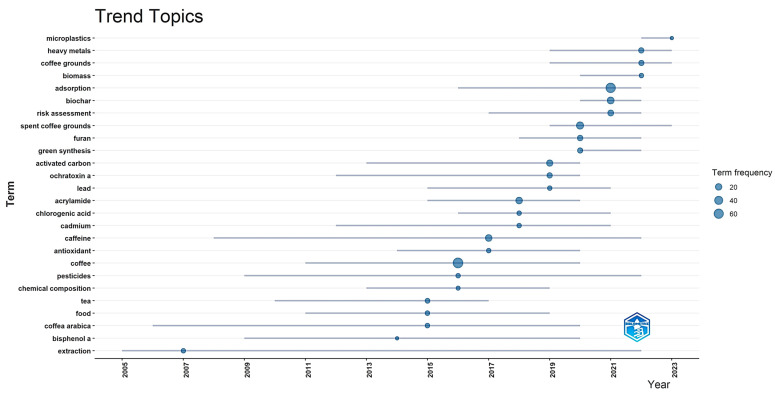
Topics studied over time in relation to chemical hazards in coffee.

The bibliometric study conducted using the RStudio program (Bibliometrix) [243] shows a change in the trends of topics studied over time, Figure 6. In the early years, topics such as the extraction of contaminants from coffee beans to the beverage, the association between coffee consumption and the risk of chronic and degenerative diseases, and the composition of coffee were the primary focus. However, since 2015, there has been a significant increase in the study of topics related to hazards (pesticides, metals, acrylamide, mycotoxins), caffeine, and antioxidant capacity. Since 2020, topics related to the potential reuse of coffee waste (due to its ability to absorb chemical hazards), risk analysis, and microplastics have been the most studied.

Based on the results of this literature review, recommendations for future research are proposed:

- Hazards in food are often present in very small concentrations, and in many research studies, the results are below the limit of detection (LOD). It is crucial to enhance analytical techniques for the rapid and accurate detection of these hazards at any point in the food chain. This is particularly important for quantifying emerging hazards, such as microplastic particles from packaging, and for testing the effectiveness of mitigation techniques.
- The vast majority of coffee-producing countries are largely dependent on coffee, so researchers need to work on improving sustainability in economic, social and environmental terms. One of the main issues with coffee processing is water pollution during wet processing. Therefore, it is essential to develop new, more environmentally friendly processing technologies and to clean up this water. Additionally, research on reusing solid waste should continue, not only to improve its absorbent capacity, but also as an important source of fibre, and for its real-world application.
- Chemical contaminants in coffee may have combined or increased effects on adverse health. It is important to conduct further research on dose-response models based on epidemiological studies, particularly for emerging hazards. This should take into account factors such as low-dose exposure, exposure to single or combined contaminants in food, and individual-related factors like race, age, and dietary habits. Additionally, it is important to use probabilistic models to address uncertainties and make informed decisions about risks.
- Hazards can arise naturally, such as metals, or be created during processing, such as acrylamide, mycotoxins, and furans. Therefore, it is essential to keep working on effective techniques to mitigate or prevent these hazards and safeguard consumer health without compromising the quality of the coffee.

## 4. Conclusions

The bibliometric study carried out on the 680 scientific articles related to the topic of chemical hazards associated with coffee between 1949 and April 2024, permits us to observe that since 2015 there has been an exponential growth in the number of research. China, Brazil and the USA were the countries with the highest production and collaboration in this field. Chemosphere, Science of the Total Environment, Food Chemistry, Journal of Agricultural and Food Chemistry, and Journal of Environmental Management were the journals with the most publications, all of them in the first quartile. Finally, the word analysis differentiated two groups: one related to the chemical hazards present in coffee and their impact on consumer health, and the other related to the waste generated from coffee and its possible reuse.

The trend analysis showed an evolution of the topics related to coffee research. Initially, there was a focus on basic research into coffee composition and extraction. This was followed by a period concentrating on the analysis and formation of chemical hazards in coffee. Finally, there has been work related to consumer health and the environment. Future research should prioritise optimising techniques for quality, safety, and sustainability.

## Figures and Tables

**Table 1:** Risk assessment publications on coffee consumption.

Hazard		Sample	Metrics				App.*	References
		Coffee	Hazard Quotient	Margin of Exposure (Equation (2))		Cancer Risk		
			(Equation (1))	Non-Neoplastic	Neoplastic	(Equation (3))		
Mycotoxins								
	AFB1	Instant	-	-	-	1.5 × 10^−9^ to 7.7 × 10^−9^	P	Taghizadeh et al., 2022 [43]
	AFB2	Instant	-	-	-	9.1 × 10^−10^ to 4.6 × 10^−9^	P	Taghizadeh et al., 2022 [43]
	AFG1	Instant	-	-	-	1.6 × 10^−9^ to 7.8 × 10^−9^	P	Taghizadeh et al., 2022 [43]
	AFG2	Instant	-	-	-	1.5 × 10^−9^ to 7.6 × 10^−9^	P	Taghizadeh et al., 2022 [43]
	OTA	Instant	2.9 × 10^−2^	8.9 × 10^3^	2.7 × 10^4^	-	D	Hoteit et al., 2024 [229]
		Instant	4.1 × 10^−1^	-	-	-	D	Foerster et al., 2021 [230]
		Instant	-	3.0 × 10^5^ to 4.7 ×10^5^	9.9 × 10^4^ to 1.1 × 10^4^	-	P	Guadalupe et al., 2024 [126]
		Classic	1.3 × 10^−1^	5.6 × 10^4^	1.7 × 10^5^	-	P	Yazdanfar et al., 2022 [231]
		Roasted	9.7 × 10^−2^	5.0 × 10^3^	1.5 × 10^4^	-	P	Yazdanfar et al., 2022 [231]
		Instant	1.2 × 10^−2^	2.8 × 10^3^	8.6 × 10^3^	-	P	Yazdanfar et al., 2022 [231]
		Roasted	3.1 × 10^−3^ to 1 × 10^−2^	-	-	-	P	Oeung et al., 2022 [232]
		Roasted	-	1.0 × 10^4^ to 1.4 × 10^4^	-	-	P	Kuiper-Goodman et al., 2010 [228]
Heavy metals								
	As	Instant	-	3.0 × 10^3^ to 3.5 × 10^3^	-	4.0 × 10^−6^ to 4.5 × 10^−6^	P	Guadalupe et al., 2024 [126]
		Green	1.5 × 10^−2^ to 5.5 × 10^−1^	1.1 × 10 to 9.1 × 10^2^	-	6.8 × 10^−6^ to 3.7 × 10^−4^	P	Guadalupe et al., 2023 [22]
		Instant	-	-	-	2.1 × 10^−8^ to 1.1 × 10^−5^	D/P	Taghizadeh et al., 2023 [24]
		Roasted	0	-	-	0	D	Kowalska, 2021 [233]
	Cd	Instant	1.4 × 10^−7^	-	-	-	P	Guadalupe et al., 2024 [126]
		Green	9.0 × 10^−4^ to 1.6 × 10^−2^	-	-	-	P	Guadalupe et al., 2023 [22]
		Instant	1 × 10^−3^	-	-	-	P	Winiarska-Mieczan et al., 2023 [25]
		Roasted	4 × 10^−3^	-	-	-	P	Winiarska-Mieczan et al., 2023 [25]
		Instant	1.4 × 10^−5^ to 2.9 × 10^−2^	-	-	-	D/P	Taghizadeh et al., 2023 [24]
		Green	2.2 × 10^−2^ to 6.7 × 10^−2^	-	-	-	P	Winiarska-Mieczan et al., 2021 [234]
		Instant	2.0 × 10^−2^	-	-	-	D/P	Khunlert et al., 2022 [28]
		Roasted	6.5 × 10^−2^ to 9.8 × 10^−2^	-	-	-	D	Kowalska, 2021 [233]
	Cr	Instant	2.5 × 10^−7^ to 2.9 × 10^−7^	-	-	-	P	Guadalupe et al., 2024 [126]
		Green	2.6 × 10^−7^ to 1.7 × 10^−5^	-	-	1.8 × 10^−7^ to 2.3 × 10^−5^	P	Guadalupe et al., 2023 [22]
		Instant	2.9 × 10^−6^ to 9.9 × 10^−6^	-	-	-	D/P	Taghizadeh et al., 2023 [24]
		Instant	3.0 × 10^−2^	-	-	-	D/P	Khunlert et al., 2022 [28]
	Cu	Instant	1.8 × 10^−5^ to 1.3 × 10^−4^	-	-	-	D/P	Taghizadeh et al., 2023 [24]
		Instant	<1.0 × 10^−2^	-	-	-	D/P	Khunlert et al., 2022 [28]
	Fe	Instant	6.9 × 10^−6^ to 8.9 × 10^−5^	-	-	-	D/P	Taghizadeh et al., 2023 [24]
		Instant	<1.0 × 10^−2^	-	-	-	D/P	Khunlert et al., 2022 [28]
	Hg	Instant	1.4 × 10^−7^	-	-	-	P	Guadalupe et al., 2024 [126]
		Green	9.0 × 10^−3^ to 1.6 × 10^−1^	-	-	-	P	Guadalupe et al., 2023 [22]
		Instant	2.9 × 10^−5^ to 5.4 × 10^−2^	-	-	-	D/P	Taghizadeh et al., 2023 [24]
		Roasted	1.1 × 10^−3^ to 2.6 × 10^−3^	-	-	-	D	Kowalska, 2021 [233]
	Ni	Instant	-	-	-	2.7 × 10^−7^ to 5.3 × 10^−5^	D/P	Taghizadeh et al., 2023 [24]
		Instant	1.0 × 10^−2^	-	-	-	D/P	Khunlert et al., 2022 [28]
	Pb	Instant	-	2.5 × 10^3^ to 6.4 × 10^3^	1.0 × 10^3^ to 2.7 × 10^3^	6.8 × 10^−9^ to 1.1 × 10^−8^	P	Guadalupe et al., 2024 [126]
		Green	-	3.5 to 1.1 × 10^2^	8.3 to 2.7 × 10^2^	4.7 × 10^−8^ to 1.5 × 10^−6^	P	Guadalupe et al., 2023 [22]
		Instant	1.5 × 10^−1^	-	-	-	P	Winiarska-Mieczan et al., 2023 [25]
		Roasted	1.4 × 10^−1^	-	-	-	P	Winiarska-Mieczan et al., 2023 [25]
		Instant	-	-	-	1.2 × 10^−10^ to 2.9 × 10^−7^	D/P	Taghizadeh et al., 2023 [24]
		Green	8 × 10^−2^ to 2.3 × 10^−1^	-	-	-	P	Winiarska-Mieczan et al., 2021 [234]
		Roasted	4.3 × 10^−3^ to 2.5 × 10^−2^	-	-	-	D	Kowalska, 2021 [233]
		Instant	4.0 × 10^−2^	-	-	-	D/P	Khunlert et al., 2022 [28]
Acrylamide		Roasted	6 × 10^−3^	-	-	5.8 × 10^−6^	D	Pekmezci y Basaran, 2024 [235]
		Instant	5.1 × 10^−3^ to 1.4 × 10^−2^	1.4 × 10^4^ to 3.6 × 10^4^	-	5.1 × 10^−6^ to 1.4 × 10^−5^	P	Guadalupe et al., 2024 [126]
		Instant	2 × 10^−4^	5 × 10^5^	-	2 × 10^−5^	D	Karami et al., 2022 [124]
		Roasted	-	7.1 × 10 to 5.2 × 10^2^	-	-	P	Claeys et al., 2016 [125]
Furan		Roasted	-	7.1 × 10^2^	1.46 × 10^4^	-	P	Cao et al., 2022 [137]
		Roasted	-	1.5 × 10^4^ to 7.4 × 10^6^	2.3 × 10^3^ to 1.3 × 10^5^	-	P	Waizenegger et al., 2012 [149]
PAH		Beans	-	9.7 × 10^5^ to 1.7 × 10^6^	-	-	P	Okaru et al., 2018 [236]
		Infusion	-	5 × 10^6^ to 6.7 × 10^6^	-	-	P	Okaru et al., 2018 [236]
		Instant	-	1.7 × 10^11^ to 4 × 10^11^	-	-	P	Taghizadeh et al., 2022 [43]
Bisphenol A		Canned	3 × 10^−2^	-	-	-	D	Lim et al., 2009 [173]
Pesticide		Roasted	<1 × 10^−1^	-	-	-	D	Radulović et al., 2024 [237]
		Instant	1.4 × 10^−9^ to 4.1 × 10^−3^	-	-	-	P	Taghizadeh et al., 2022 [43]

* App. = Approach; P = Probabilistic; D = Deterministic.

## Data Availability

The original contributions presented in the study are included in the article and Supplementary Material, further inquiries can be directed to the corresponding authors.

## Supplementary Materials

The following supporting information can be downloaded at: https://www.mdpi.com/article/10.3390/toxics12070526/s1, Figure S1: Total scientific output by country; Figure S2: Main journals with publications related to the topic; Figure S3: Collaboration of authors; Figure S4: Three-Field Plot. AU_UN: Author’s Affiliation, AU_CO: Author’s Country, AU: Author.
