# Supplementary material for: A Comprehensive Bibliometric Study in the Context of Chemical Hazards in Coffee
Source: Toxics. 2024 Jul 22;12(7):526. doi: 10.3390/toxics12070526 (PMC11281111; doi:10.3390/toxics12070526)
Supplement: Supplementary file 1 [file toxics-12-00526-s001.zip › toxics-3086600-supplementary.pdf]

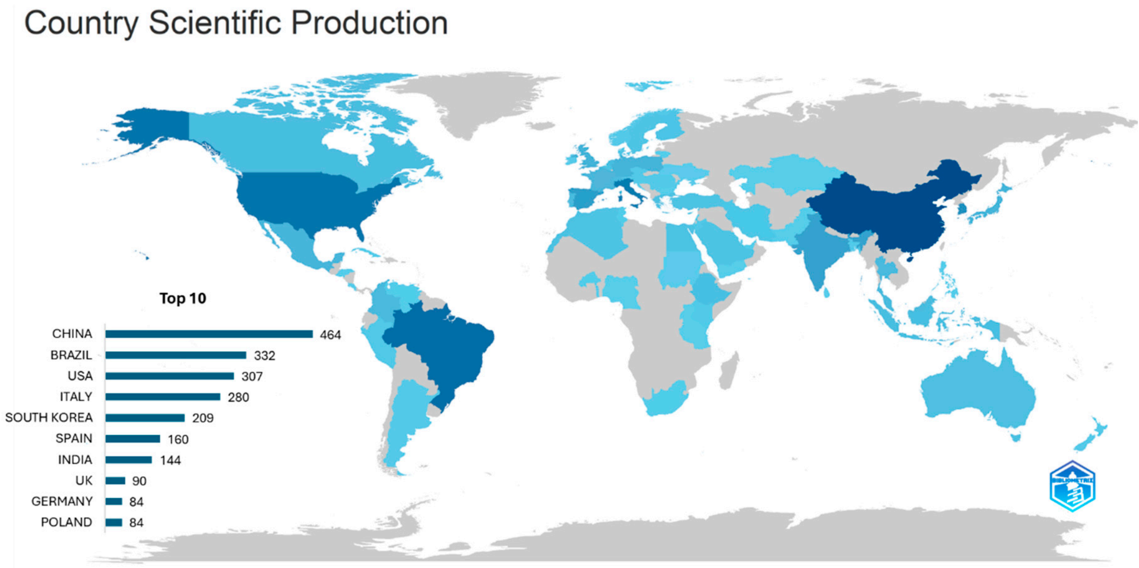

Figure S1. Total scientific output by country.

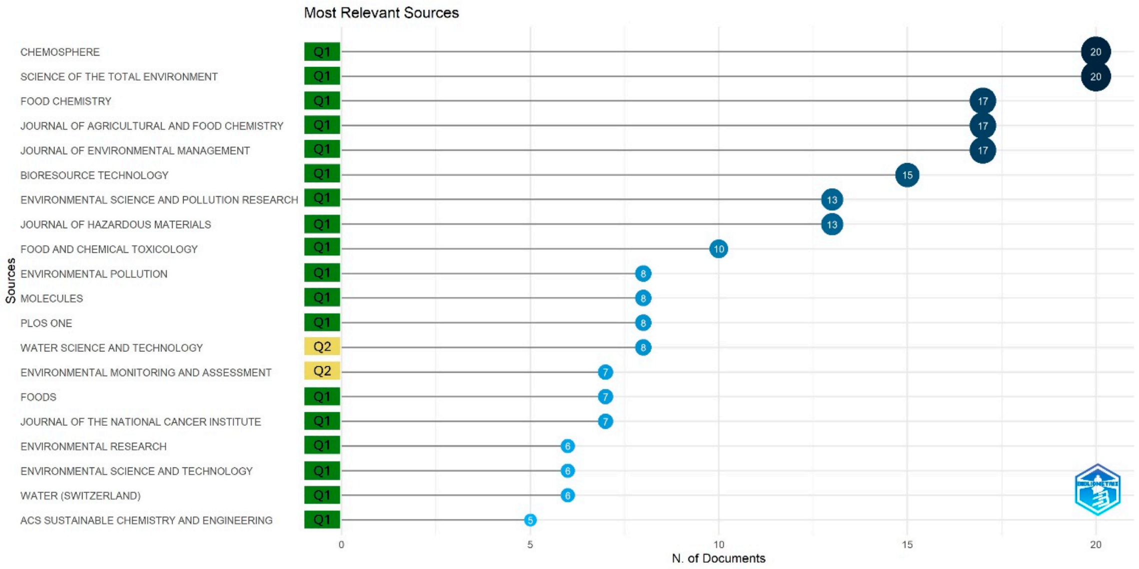

Figure S2. Main journals with publications related to the topic.

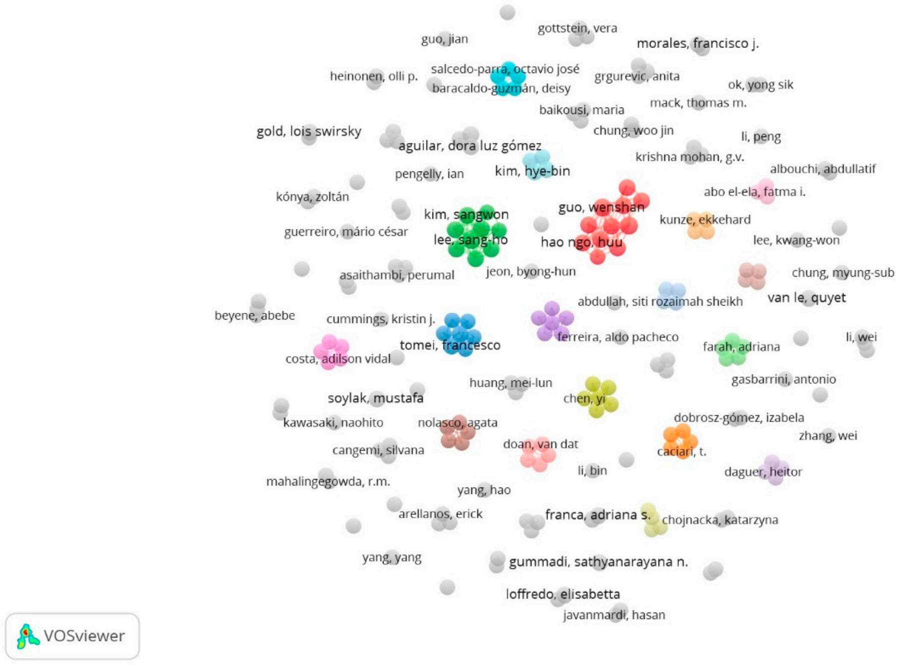

Figure S3. Collaboration of authors.

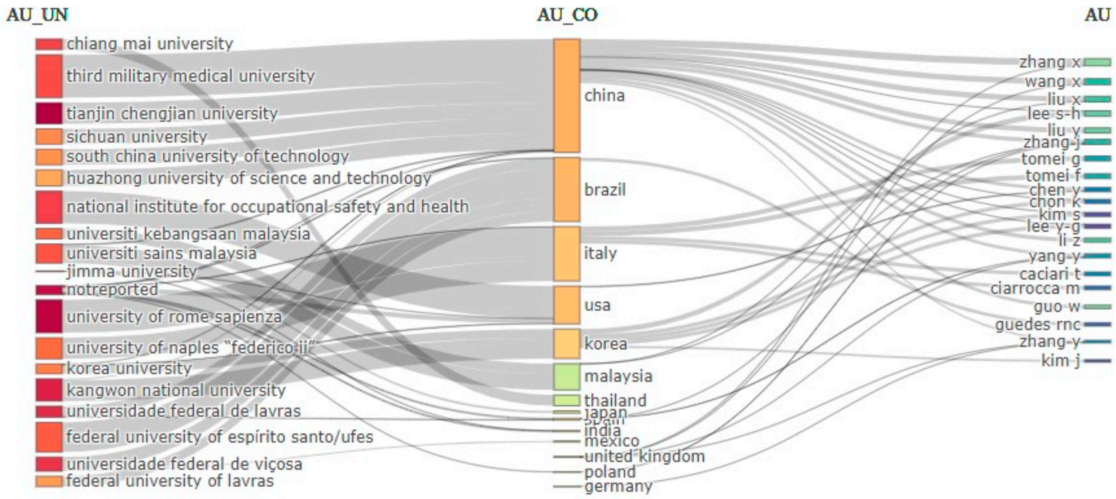

Figure S4. Three-Field Plot. AU_UN: Author's Affiliation, AU_CO: Author's Country, AU: Author.
